# A Pilot Study for Return of Individual Pharmacogenomic Results to Population-Based Cohort Study Participants

**DOI:** 10.31662/jmaj.2021-0156

**Published:** 2022-03-11

**Authors:** Kinuko Ohneda, Masahiro Hiratsuka, Hiroshi Kawame, Fuji Nagami, Yoichi Suzuki, Kichiya Suzuki, Akira Uruno, Mika Sakurai-Yageta, Yohei Hamanaka, Makiko Taira, Soichi Ogishima, Shinichi Kuriyama, Atsushi Hozawa, Hiroaki Tomita, Naoko Minegishi, Junichi Sugawara, Inaho Danjoh, Tomohiro Nakamura, Tomoko Kobayashi, Yumi Yamaguchi-Kabata, Shu Tadaka, Taku Obara, Eiji Hishimuma, Nariyasu Mano, Masaki Matsuura, Yuji Sato, Masateru Nakasone, Yohei Honkura, Jun Suzuki, Yukio Katori, Yoichi Kakuta, Atsushi Masamune, Yoko Aoki, Masaharu Nakayama, Shigeo Kure, Kengo Kinoshita, Nobuo Fuse, Masayuki Yamamoto

**Affiliations:** 1 Tohoku Medical Megabank Organization, Tohoku University, Sendai, Japan; 2 Advanced Research Center for Innovations in Next-Generation Medicine, Tohoku University, Sendai, Japan; 3 Department of Pharmacotherapy of Life-Style Related Diseases, Graduate School of Pharmaceutical Sciences, Tohoku University, Sendai, Japan; 4 Faculty of Pharmaceutical Sciences, Tohoku University Hospital, Sendai, Japan; 5 Department of Clinical Genetics, Jikei University Hospital, Tokyo, Japan; 6 Department of Clinical Genetics, Ageo Central General Hospital, Ageo, Japan; 7 International Research Institute of Disaster Science, Tohoku University, Sendai, Japan; 8 The United Centers for Advanced Research and Translational Medicine, Tohoku University Graduate School of Medicine, Sendai, Japan; 9 Environment and Genome Research Center, Tohoku University Graduate School of Medicine, Sendai, Japan; 10 Department of Obstetrics and Gynecology, Tohoku University Graduate School of Medicine, Sendai, Japan; 11 Department of Pediatrics, Tohoku University Graduate School of Medicine, Sendai, Japan; 12 Department of Otolaryngology-Head and Neck Surgery, Tohoku University Graduate School of Medicine, Sendai, Japan; 13 Division of Gastroenterology, Tohoku University Graduate School of Medicine, Sendai, Japan; 14 Department of Medical Genetics, Tohoku University Graduate School of Medicine, Sendai, Japan; 15 Department of Medical Informatics, Tohoku University Graduate School of Medicine, Sendai, Japan

**Keywords:** Return of genomic results, Pharmacogenomics, Population-based cohort study

## Abstract

**Introduction:**

Pharmacogenomic (PGx) testing results provide valuable information on drug selection and appropriate dosing, maximization of efficacy, and minimization of adverse effects. Although the number of large-scale, next-generation-sequencing-based PGx studies has recently increased, little is known about the risks and benefits of returning PGx results to ostensibly healthy individuals in research settings.

**Methods:**

Single-nucleotide variants of three actionable PGx genes, namely, *MT-RNR1*,* CYP2C19*, and *NUDT15*, were returned to 161 participants in a population-based Tohoku Medical Megabank project. Informed consent was obtained from the participants after a seminar on the outline of this study. The results were sent by mail alongside sealed information letter intended for clinicians. As an exception, genetic counseling was performed for the *MT-RNR1* m.1555A > G variant carriers by a medical geneticist, and consultation with an otolaryngologist was encouraged. Questionnaire surveys (QSs) were conducted five times to evaluate the participants’ understanding of the topic, psychological impact, and attitude toward the study.

**Results:**

Whereas the majority of participants were unfamiliar with the term PGx, and none had undergone PGx testing before the study, more than 80% of the participants felt that they could acquire basic PGx knowledge sufficient to understand their genomic results and were satisfied with their potential benefit and use in future prescriptions. On the other hand, some felt that the PGx concepts or terminology was difficult to fully understand and suggested that in-person return of the results was desirable.

**Conclusions:**

These results collectively suggest possible benefits of returning preemptive PGx information to ostensibly healthy cohort participants in a research setting.

## Introduction

Individual genomic data collected in large-scale cohort studies are expected to be used as a tool for improving disease treatment and prevention and the management of personal healthcare. Pharmacogenomics is the study of individual genetic variability and how it influences drug response. The results of pharmacogenomic (PGx) testing provide valuable information on drug selection and appropriate dosing, maximization of efficacy, and minimization of adverse effects ^(1), (2)^. However, several considerations were raised when returning PGx results to individuals ^(3)^. For instance, the Industry Pharmacogenomics Working Group published “Points-to-Consider” related to the return of genomic results in the context of drug development ^(4), (5)^. Most concerns, including those related to ethical and legal considerations, clinical relevance, data quality control, and reporting results to participants and clinicians, are also applied in nonindustrial research and clinical settings. Although the number of large-scale, next-generation-sequencing-based PGx studies has increased [e.g., the Electronic Medical Records and Genomics Network (eMERGE-PGx) study ^(6)^], little is known about the risks and benefits of returning PGx results to study participants. Moreover, potential differences between clinical and research settings might affect the outcome and impact on participants. In a clinical setting, patients are aware of the practical benefits of PGx testing as the results can be utilized by clinicians immediately after the genetic testing. On the contrary, the situation is different in returning preemptive PGx results in research settings ^(7), (8), (9)^. Population-based cohort study participants might not perceive any practical benefits from their PGx results. In addition, regardless of the elapsed time, patients should be informed of the PGx testing results by physicians so as to specify the correct treatment, thereby enabling patients to remember their relevance and the potential use of this data. Therefore, the potential benefits and challenges of returning PGx results should be evaluated.

The Tohoku Medical Megabank (TMM) project is part of a national project established in 2012 at Tohoku University and Iwate Medical University. We have conducted two large-scale, prospective cohort studies (the TMM Project Community-based Cohort Study and the TMM Project Birth and Three-generation Cohort Study) and developed an integrated biobank comprising biospecimens, multiomics data including whole-genome sequencing (WGS) data, and health information ^(10), (11), (12), (13), (14), (15), (16), (17)^. By using the WGS data, we accurately designed a system to return individual genomic results (return of genomic results: ROGR) to benefit the study participants for their personal healthcare management. Since the ROGR studies on ostensibly healthy individuals had been rarely conducted, we planned a series of pilot studies in a stepwise manner. The first pilot study was conducted to return genetic results associated with familial hypercholesterolemia to individuals with dyslipidemia. Since the participants had received their blood lipid data, receiving individual genomic information could be acceptable. A total of 215 individuals consented to participate in the study, of whom 23 were pathogenic variant carriers. This study was performed as the second pilot study. Preemptive PGx genotyping was performed for the study participants. In the third study, it was planned to return genomic information related to single-gene disorders with intermediate penetrance and/or adult-onset phenotype. The details of the TMM project ROGR pilot study are described elsewhere ^(18)^.

In the present study, we report our experience in returning individual PGx results to population-based genome cohort participants enrolled in the TMM project. In addition, we demonstrate that most of the participants felt that they could acquire basic PGx knowledge sufficient to understand their genomic results and were satisfied with their potential benefit and use in future prescriptions. These results suggest possible benefits of returning PGx results in a research setting.

## Materials and Methods

### The execution organization of the ROGR pilot study

All studies were conducted in accordance with the “Ethical guidelines for human genome and gene analysis research” presented by the Ministry of Education, Culture, Sports, Science and Technology (MEXT), Ministry of Health, Labor and Welfare (MHLW), and Ministry of Economy, Trade and Industry, as well as with the “Ethical guidelines for medical and health research involving human subjects” presented by MEXT and MHLW. The PGx pilot study was approved by the ToMMo Research Ethics Review Board of Tohoku University (approval code: 2019-4-094), and the study plan was reviewed by the Return of Genomic Results Review Committee (RGRRC).

### Information to stakeholders

Prior to the recruitment of the participants, the study plan was announced to physicians and pharmacists working in the Miyagi Prefecture as stakeholders for the study. The Pharmaceutical Department of Tohoku University Hospital (TUH) supported clinicians prescribing based on PGx results and assisting ToMMo staff in responding to local clinician inquiries. Furthermore, we held oral briefings at academic conferences for community and hospital pharmacists to inform the scientific community about the study and request for their cooperation. For local clinicians, the study announcement and request for cooperation were made using a mailing list. In addition, we prepared a leaflet targeting local clinicians in the form of this study and made it available at various academic conferences.

### Detection of individual genotypes and validation

**Figure 1.**
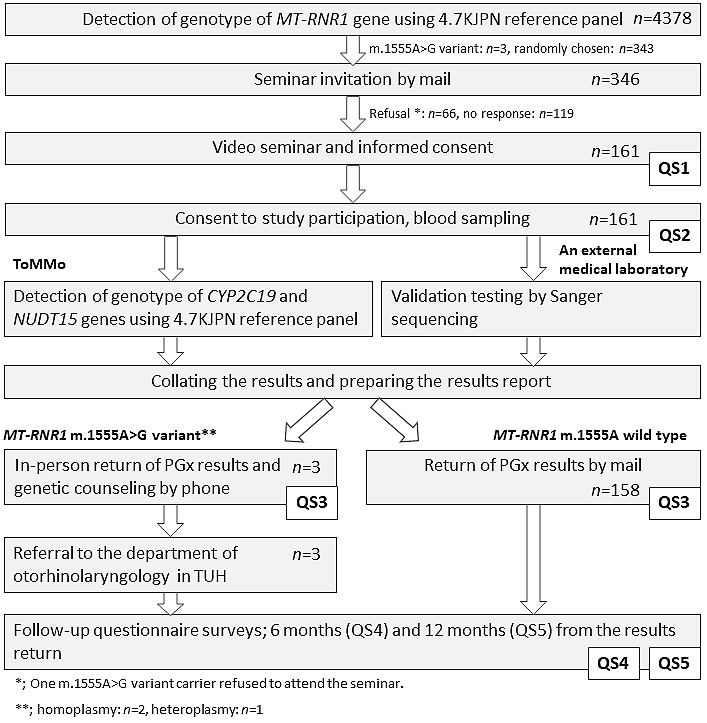
Flow diagram of the study design. The flow diagram of the study and the number of study participants are shown. QS: Questionnaire survey, ToMMo: Tohoku Medical Megabank Organization, TUH: Tohoku University Hospital.

The flow diagram of the study design is presented in Figure 1. The individual IDs for the participants were securely converted into internal IDs for anonymized data ^(18)^, and two bioinformaticians dealing only with anonymized data independently performed variant detection using the WGS data. In the TMM project, WGS of the cohort participants was performed, and genome reference panels for the Japanese population were constructed. The genome reference panel has been updated with an increased number of participants with the WGS data (1KJPN, 2KJPN, 3.5KJPN, 4.7KJPN, and 8.3KJPN; Allele Frequency Panels)^(19), (20)^. The present study used 4.7KJPN constructed from 4,773 Japanese individuals, of whom 4,378 were TMM cohort study participants.

To detect individual genotypes, the pathogenic variant of the mitochondrial 12S ribosomal RNA, encoded by *MT-RNR1* m.1555A>G [pathogenic variant (rs267606617), hereafter referred to as “m.1555A>G”], was searched against the file of the merged individual genotype of 4.7KJPN. Three participants were identified as carriers of the m.1555A>G variant and were recruited for the study. We evaluated the presence of the *CYP2C19* variant *1-*3 (*1: wild type; *2: c.681G>A (p.Pro227=), rs4244285; and *3: c.636G>A (p.Trp212Ter), rs4986893) and *NUDT15* codon 139 [c.415C>T (p.Arg139Cys), rs116855232; c.416G>A (p.Arg139His), rs147390019] only in the cohort participants that consented to participate in the study (*n* = 161). The participant genotypes were securely transferred to medical genetics experts through ID conversion. To validate the genomic results, the blood samples of the participants were sent to LSI Medience Corporation (LSI), a registered clinical laboratory that meets the standard policy by the Regulation for Enforcement of the Act on Clinical Laboratory Technicians governed by the MHLW. The results from LSI were collated with the ToMMo data. The m.1555G allele of a m.1555A>G heteroplasmic variant could not be detected using a variant panel of fixed genotypes for mitochondrial DNA, as the proportion of variant copies was <20% in this individual. The variant was detected by Sanger sequencing *via* LSI, which was confirmed by inspecting the proportion of sequence reads with the variant in the WGS data in ToMMo and further checked *via* LSI using the restriction fragment length polymorphism methods.

### Mental healthcare for the participants and evaluation of psychological impacts

To evaluate the practical benefits to and psychological impact on the participants following the return, we conducted a series of QSs. In Table 1, the survey items are listed. We used three kinds of psychological testing: the Japanese version of the Kessler Psychological Distress Scale (K6) ^(21)^, the Japanese translation of the Profile of Mood States second edition ^(22)^, and the Japanese-language version of the Impact of Event Scale-Revised ^(23)^. The results of time-dependent changes in the psychological effects on the participants will be described separately. Eventually, four of nine subjects with high K6 scores underwent psychological assessment, although none left the study prematurely due to mental health problems.

**Table 1.** Questionnaire Items.

Timing of survey	QS1	QS2	QS3	QS4	QS5
	Seminar invitation	Informed consent	Return of genomic results	6 months from QS3	12 months from QS3
K6	●	●	●	●	●
POMS-2		●	●	●	●
IES-R			●	●	●
Knowledge about PGx	●	●	●		
Intent of participation	●			●	
Satisfaction with participation			●	●	●

## Results

### Selection of variants in the three PGx genes for return in the pilot study

The PGx genes for return were selected according to the following criteria: (i) there was clear evidence of the genotype-phenotype associations for Japanese individuals; (ii) the results were clinically actionable; (iii) the frequency of the mutant alleles was not rare in the Japanese population; and (iv) the target drug was commonly prescribed in Japan. First, we examined the levels of evidence, guidelines, and actionability using PharmGKB (https://www.pharmgkb.org/), the Table of Pharmacogenomic Biomarkers in Drug Labeling by the US Food and Drug Administration (https://www.fda.gov/drugs/science-and-research-drugs/table-pharmacogenomic-biomarkers-drug-labeling), and Clinical Pharmacogenetics Implementation Consortium guidelines (https://cpicpgx.org/). Subsequently, we examined data from Japanese patients and selected three genes, namely, *MT-RNR1*, *CYP2C19*, and *NUDT15* (Table 2). Drugs whose efficacy and safety are affected by the genotype (hereafter referred to as target drugs) are listed in Table 2. A previous study reported that the m.1555A>G pathogenic variant of *MT-RNR1* in 1.9% of Japanese hearing-impaired patients (*n* = 264) diagnosed using the invader-based genetic screen test ^(24)^, whereas another report described that 1 in 1,683 (0.06%) individuals from the Japanese general population participating in the IWAKI Health Promotion Project was found to harbor the m.1555A>G variant according to TaqMan genotyping ^(25)^. Therefore, we searched this variant against the file of the merged individual 4.7KJPN genotype, which resulted in the recruitment of three variant carriers for the study. We selected the *CYP2C19* polymorphism based on its serving as a predictive marker of the effectiveness of proton pump inhibitors (PPIs), lansoprazole, and omeprazole and because PPI-mediated eradication of *Helicobacter pylori* is a standard and preventive treatment for gastric cancer in Eastern Asia ^(26), (27)^. The Cys/Cys genotype of *NUDT15* codon 139, which predicts the risk of thiopurine-induced severe adverse effects, is observed in ~1% of the Japanese population ^(28), (29), (30)^.

**Table 2.** Genes and Drugs Eligible for Disclosure.

Gene	Drug	CPIC guidelines	CPIC level	PharmGKB level of evidence	FDA-approved drug label
*MT-RNR1* m.1555	Aminoglycosides				
*CYP2C19*	Clopidogrel	Yes	A	1A	Actionable
	Voriconazole	Yes	A	1A	Actionable
	Lansoprazole	Yes	A	1A	Informative
	Omeprazole	Yes	A	1A	Actionable
*NUDT15*	Azathioprine	Yes	A	1A	Testing recommended
	Mercaptopurine	Yes	A	1A	Testing recommended

Level Definitions for the CPIC Genes/Drugs are described in https://cpicpgx.org/prioritization/#flowchart. Clinical annotation levels of evidence for Pharm GKB are described in https://www.pharmgkb.org/page/clinAnnLevels.

### Recruitment and sampling of research participants

A total of 346 primary participants recruited for the study were randomly selected from 4,378 cohort participants with the WGS data. All primary participants (i.e., three carriers of the m.1555A>G variant and 343 randomly chosen TMM cohort participants) were >20 years of age, with an average age of 61.3 and 64.3 years for study and nonstudy participants, respectively. The age and sex distributions of the participants are presented in Table 3. A recruitment letter was sent with information regarding the ROGR pilot study and a request for them to attend a video seminar. A total of 161 primary participants agreed to participate. A video seminar about the study was held for ~10 participants at a time in seven local assessment centers located in the Miyagi Prefecture ^(14)^. At the time of enrollment, none of the participants have taken the target drugs described in this study. Of the 161 seminar participants, 70 were male and 91 were female. Among the three subjects carrying the m.1555A>G variant, one did not consent to attend the seminar. The participants were asked to bring their prescription record notebook, a commonly used record of personal prescription history in Japan. At the seminar reception, the community support center staff interviewed the participants about their medical history.

**Table 3.** Age and Sex Distribution.

Age group	Male	Female
20–29	0	0
30–39	7	8
40–49	8	8
50–59	12	19
60–69	14	23
70–79	23	30
80–89	6	2
90–99	0	1
Total	70	91

### Video seminar and informed consent (IC)

The seminar participants were shown a video describing basic PGx concepts and the outline of this study for 10 min. Subsequently, a clinician provided supplementary information and answered questions raised by the seminar participants. Written informed consent was obtained from the seminar participants by the genome medical research coordinators, who underwent specialized training through ToMMo ^(31)^. All the seminar participants consented to participate in the study. Whole blood samples were taken from the participants and sent to an external registered clinical laboratory to conduct PGx gene sequencing so as to confirm the WGS results.

### Return of the genomic results

**Figure 2.**
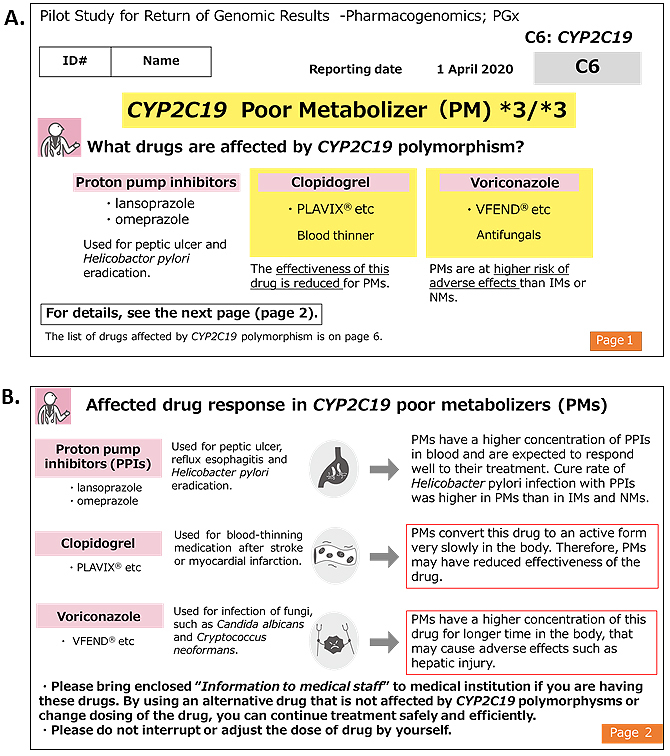
Results report for research participants sent *via* mail. English translation of representative results reports for participants carrying a CYP2C19 variant [poor metabolizer (PM)]. A, Page 1 (of 6 pages) of the report shows drugs that can be influenced by the presence of specific CYP2C19 polymorphisms. B, Page 2 of the results report shows detailed medicinal information and drug response with regard to metabolism status. Participants were advised to take the enclosed information to healthcare professionals, as necessary.

**Figure 3.**
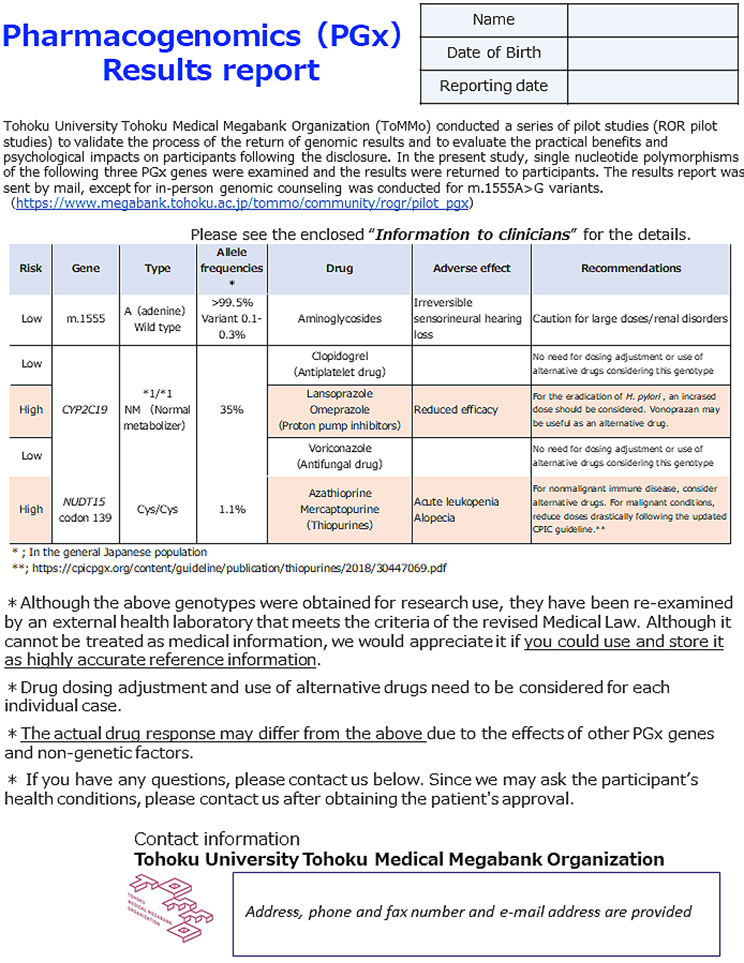
Summary results report for clinicians. English translation of the representative results. The PGx results for genes are given in a table along with prescription recommendations. Information about the pilot study is included and accessible through the provided URL link along with the contact information included at the bottom of the report. Contact information is hidden for the manuscript.

The genomic testing results provided by a registered clinical laboratory were collated with individual WGS data stored in the ToMMo. All the results matched, except for one case of a heteroplasmic m.1555A>G variant. The genotype results of the participants are presented in Table 4. The allele frequencies of *CYP2C19* ^(32), (33)^ and *NUDT15* ^(29)^ were consistent with those previously reported in Japanese individuals (ToMMo 4.7KJPN and a previous report ^(20)^). The results report was sent to the participants by mail. Two types of reports were enclosed: one intended for the study participants and the other for healthcare professionals. The latter was prepared using two sets of sealed documents. Representative reports for the participants and healthcare professionals are presented in Figure 2 and 3, respectively. In the reports for the participants, the generic and brand names of the drugs considered as potential risks alongside their indication, medicinal effects, and individual predicted genotype-based response to the drug were informed. We asked the participants to bring the enclosed “information for healthcare professionals” to a hospital or pharmacy for any necessary treatment revision and to not interrupt or change the dose of the drug at their own discretion. In addition, we noted that drug-response prediction might not always be accurate due to individual variations in other PGx genes, and several nongenetic factors, such as food intake, liver and kidney functions, and concomitant drugs, could potentially affect drug response. Individual reports for healthcare professionals were prepared as a single sheet containing all three genes, which makes them manageable as supporting medical information. These reports include medication and dosage recommendations based on the genomic results. In addition to the results report, information on the ROGR pilot studies, PGx results interpretation, and a URL linked to PGx guidelines and databases were enclosed. The participants could request additional sets of “information for healthcare professionals” as needed and contact the researcher for additional information.

**Table 4.**
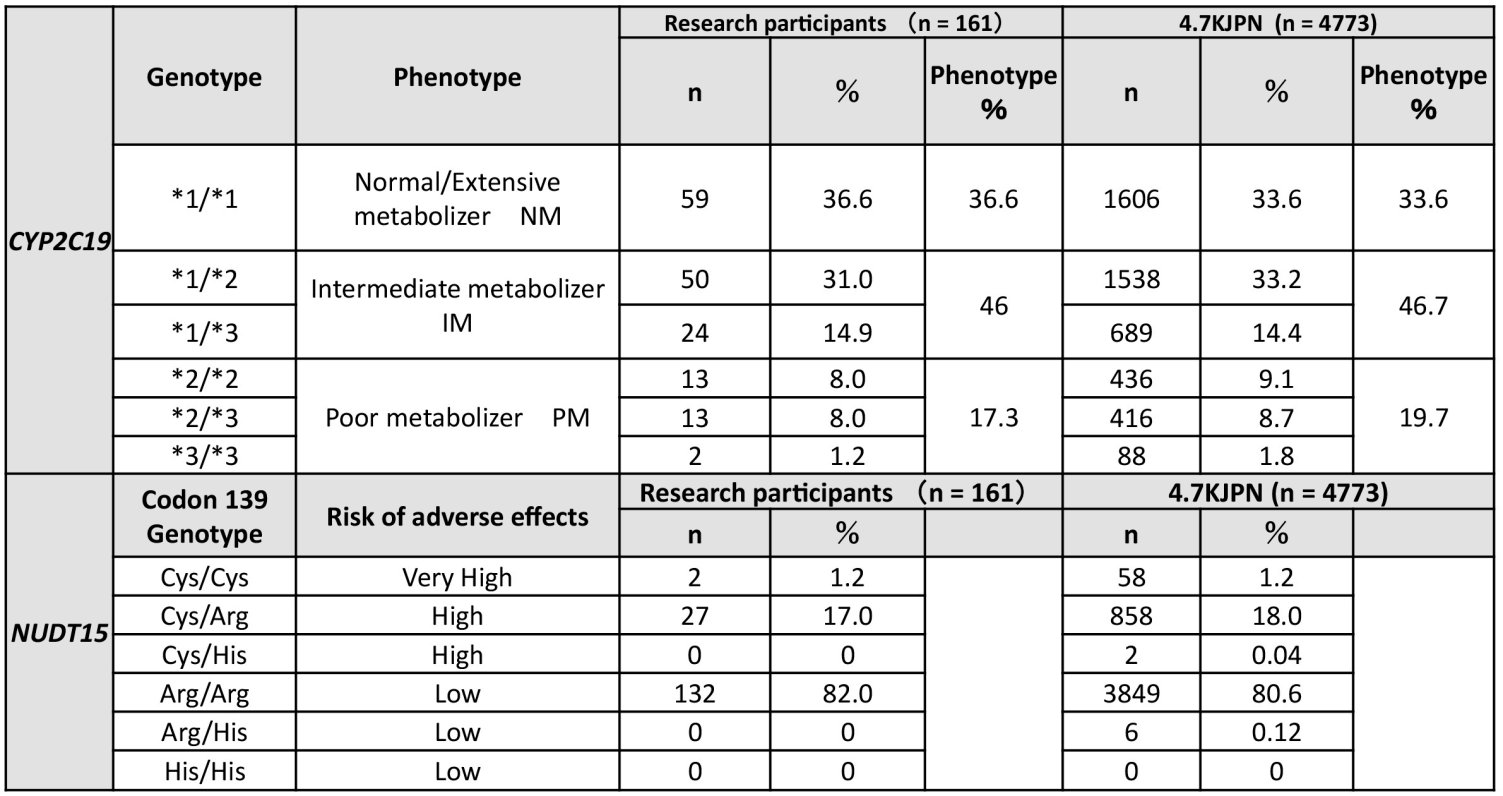
Genotyping Results of PGx Testing for the *CYP2C19* and *NUDT15* Genes.

### Return of the PGx results and genetic counseling to carriers of the m.1555A>G variant

As described in the Methods section, one participant harbored a heteroplasmic m.1555A>G variant with m.1555A as the dominant type. Because the heteroplasmic variant frequency varies according to tissue type ^(34)^, we returned the genomic results individually according to the same flow employed for carriers of the homoplasmic m.1555A>G variant.

Due to COVID-19 pandemic constraints, genetic counseling and return of the PGx test results for the carriers of the m.1555A>G variant were performed by phone, with the results report sent via mail in advance. In addition to the information on the drug-induced hearing loss, the results report included an example family tree demonstrating maternal inheritance and a list of aminoglycosides used in Japan. In addition, two sets of a medication alert card for aminoglycosides in Japanese and English were provided. Prior to genetic counseling, we informed the participants that a family history of hearing loss would be discussed. Genetic counseling was performed by a medical geneticist. The profile of the m.1555A>G variant carriers is presented in Table 5. They were all females in the 70s. Case 1 has a hearing loss in her left ear that occurred in her 40s, although her medication history of aminoglycosides is uncertain. Case 2 does not have a hearing impairment. Both cases have siblings diagnosed with drug-induced hearing loss without genetic testing. Case 3 is a carrier of the heteroplasmy variant who does not have past medical nor family history of hearing impairment. After ~30 min of genetic counseling performed by a clinical geneticist, all the participants carrying the m.1555A>G variant (*n* = 3; two homoplasmic and one heteroplasmic) consented to visit an otolaryngologist in TUH, for which we prepared a medical information provision form with the results of the PGx testing and a genetic family tree. Since all of them shared the results with relatives who are at risk, we informed them that they could visit TUH and have a genetic counseling with an otolaryngologist. After the examination, the attending otolaryngologist entered aminoglycosides as contraindicated drugs in the participant electronic healthcare records (EHRs) in TUH.

**Table 5.** Profiles of the *MT-RNR1* m.1555A > G Variant Carriers.

Case ＃	Age	Sex	Genotype	Presence of hearing loss	
				Study participant	Families
**1**	70s	Female	Homoplasmy	Yes	Yes
**2**	70s	Female	Homoplasmy	No	Yes
**3**	70s	Female	Heteroplasmy	No	No

### Questionnaire survey results

**Figure 4.**
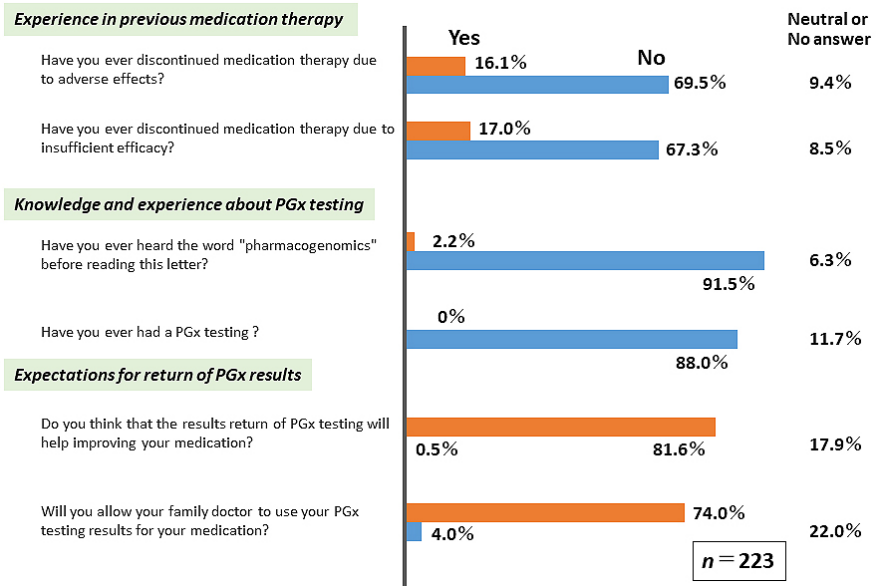
Questionnaire survey results before returning the genomic results. The questionnaire survey results regarding past experience and problems with medication, knowledge of PGx, and expectations of PGx results are shown. The questionnaire was administered during the study recruitment phase, and answers were obtained from 223 participants, including seminar non-attendants.

**Figure 5.**
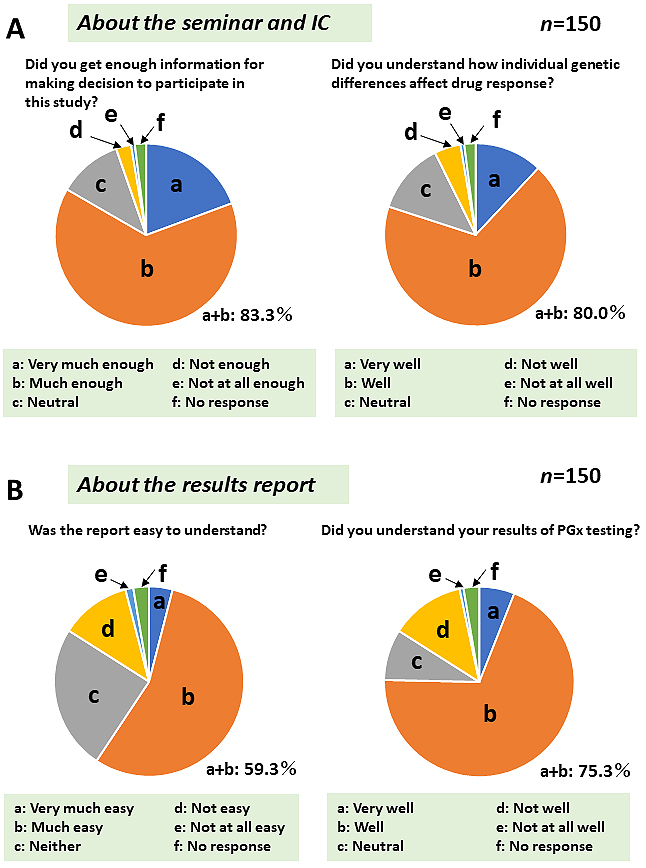
Questionnaire survey results after returning the genomic results. The questionnaire survey results regarding seminar and IC (A) and the results report (B) content comprehension are presented. The boxes at the bottom of each pie chart show the survey choices (a-f). Answers were obtained from 150 subjects who participated in the study. The percentage of the participants choosing answers A or B is shown at the bottom right.

Because detailed information regarding the PGx field is not generally accessible to a broader audience, we provided participants with basic PGx information several times throughout the study period. Questionnaire surveys (QSs) were conducted five times before (QS1 and QS2, Figure 4) and after (QS3, QS4, and QS5, Figure 5) the ROGR. The results indicated that only a small percentage of participants (<17%) had previous problems with medication therapy. As expected, majority of the participants were unfamiliar with the term PGx, and none had undergone PGx testing before the study. However, after reading the recruitment letter, >70% of the participants expected that the PGx testing results might be beneficial (Figure 4). Upon receiving the PGx results, majority (>80%) of the participants felt that they could obtain sufficient information in the seminar and the IC form (Figure 5A). However, only a small percentage of the participants felt that the results report was easy to understand, whereas the majority reported having only a moderate understanding of the PGx testing results (Figure 5B). In the free description in QS4, some participants suggested that in-person return of the results was desirable. These results indicate a limitation in the participants’ comprehension of the returned genomic results without an in-person interaction.

## Discussion

We conducted a pilot study with regard to the return of three PGx results to participants enrolled in a large-scale genome cohort TMM project. We identified three carriers of the m.1555A>G variant, and they were subsequently referred to the otorhinolaryngology department in TUH for further testing and counseling. Genetic counseling was successfully performed by a clinical geneticist by phone in addition to return of the results report prior to genetic counseling. Most of the participants reported that their knowledge and understanding of the PGx testing have improved after receiving the results report. Given that large-scale genome cohort studies have been increasingly conducted for healthy individuals in research settings, this study provides key insights into the potential use of PGx information in personalized healthcare management.

However, some challenges and limitations remain. First, we could only return a limited number of PGx genes. We considered the inclusion of the *CYP2D6* polymorphism, as this gene predicts the risk of severe adverse effects when using codeine ^(35)^; however, it was excluded, because the number of carriers with a higher risk of poor treatment outcomes was much lower in individuals with Japanese rather than European ancestry ^(36)^. In addition, genotype confirmation is difficult for such a large number of subjects and would merit a separate study to better focus on participant education with regard to this specific gene. To scale up the number of PGx genes and participants, the cost-effectiveness of preemptive PGx testing should be evaluated and the scalability of human resources enhanced. Second, we encountered challenges in the process of confirming the m.1555A>G heteroplasmic variant, as described in the Methods section. We could detect the genotype by collating the genomic results independently obtained by two different laboratories. This suggests the importance of the validation testing because mismatch of the genotyping results could also occur due to human error during handling of the samples. Third, as reported in the Return of Actionable Variant Empirical (RAVE) study conducted at the Mayo Clinic ^(37)^, we encountered participant contact-related challenges. Approximately 33% (*n* = 119) of the 346 subjects invited to participate in the video seminar did not respond to the invitation letter; thus, a second invitation was sent, followed by attempts to contact the subjects by phone. We needed to call more than three times to contact some subjects who responded that they would attend the seminar. These experiences indicate that a considerable amount of time and human resources are required for successful participant recruitment. Fourth, even though the basic information of PGx had been given several times, some participants felt that the PGx concepts or terminology was difficult to fully understand. To accurately evaluate and take measures for improvement of the participants’ understanding, objective testing is necessary.

Although this study showed possible benefits of the return of PGx results individually in a research setting, further challenges and limitations hinder clinical implementation in Japan. In Japanese medical institutions, PGx testing is conducted in patients with an existing disease diagnosis. However, the Japanese health insurance currently covers PGx testing for only two genes, namely, the *UGT1A1* polymorphism, which predicts the risk of severe adverse effects associated with the administration of irinotecan ^(38)^, and the *NUDT15* polymorphism, which was examined in this study. We added the health insurance information in the results report for a *NUDT15* Cys/Cys variant, because the insurance coverage began in February 2019 during this study*.* With regard to the m.1555A>G variant data, medical implementation was performed by entering the contraindicated drug information in the appropriate EHRs of TUH. Nevertheless, the Japanese EHR system is currently under development to include the use of the PGx data. To clinically implement PGx data considerations for a large number of undiagnosed patients, a multicenter study, such as the one employed to design the eMERGE-PGx study ^(6), (39), (40)^, needs to be conducted in the near future. Because the TMM project was conducted involving population-based cohort participants, the PGx results must be submitted separately to healthcare professionals. Therefore, we prepared two sets of sealed documents with contact information. To improve information accessibility, it would be better to include the results of the Miyagi Medical and Welfare Information Network (MMWIN), a medical network system that provides data storage services for medical and pharmaceutical institutes ^(41)^. This system was established in 2013 to protect medical data from destruction by natural disasters, such as the Great East Japan Earthquake. Because MMWIN enables sharing of patient information, including contraindications, entry of such information by healthcare professionals could potentially help in preventing adverse drug effects. An update and further expansion of the information provided to users are worthy of further consideration to promote personalized medicine.

In summary, we described the possible benefits and challenges of returning PGx results *via* a large-scale integrated research biobank. The limitations experienced in conducting this research might help in the planning of other ROGR studies. The practical effectiveness of returning PGx data needs to be further evaluated.

## Article Information

### Conflicts of Interest

None

### Sources of Funding

This work was supported by the MEXT TMM project and the Japan Agency of Medical Research Development [AMED, JP 20 km0105001, and JP 20 km0105002].

### Acknowledgement

We thank the chairperson Yoshimitsu Fukushima and members of the RGRRC for their critical comments and suggestions, Drs. Akimune Fukushima and Tomoharu Tokutomi for their collaboration with the ROGR pilot study, and Drs. Shin-ichi Usami and Shin-ya Nishio for their valuable suggestions regarding the m.1555A>G variant. We also thank Dr. Evelyn Marie Gutiérrez Rico and Editage (www.editage.com) for English language editing, Miho Kuriki for figure illustration, and Michiko Sugawara and Yoko Haga for secretarial assistance. The list of participating ToMMo members is available at https://megabank.tohoku.ac.jp/english/a201201.

### Author Contributions

Conceptualization: K.O., M.H., H.K., F.N., N.F., M.Y. Data Curation: S.O., I.D., T.N., M.N. Formal analysis: Yo.S., S.T., T.O., E.H., Y.Y-K. Funding acquisition: M.Y. Investigation: K.S., A.U., M.S-Y., M.M., Yu.S., M.N., M.T., H.T., Y.Ho., J.S., Yo K., Methodology: M.H., F.N. Project administration: K.O., M.H., H.K., N.F. Resources: S.K., A.H., N.M., J.S., T.K. Supervision: M.S., N.M., Yu K., A.M., Y.A., S.K., K.K., M.Y. Validation: Y.Y-K., S.T. Visualization: K.O., M.H., F.N., Y.Ha. Writing―original draft: K.O. Writing―review & editing: K.O., M.H., H.K., F.N., Yo.S., N.F., Y.Y-K., Y.Ho., Yo K., M.N., M.Y.

### Approval by Institutional Review Board (IRB)

The project protocols were reviewed and approved by the Ethics Committee of Tohoku University Tohoku Medical Megabank Organization (IRB approval code number: 2019-4-094).
